# Edge Control in the Computer-Controlled Optical Surface

**DOI:** 10.3390/mi12101154

**Published:** 2021-09-25

**Authors:** Lianmin Yin, Hao Hu, Chaoliang Guan, Yifan Dai, Zelong Li

**Affiliations:** 1 Laboratory of Science and Technology on Integrated Logistics Support, College of Intelligence Science and Technology, National University of Defense Technology, Changsha 410073, China; yinlianmin18@nudt.edu.cn (L.Y.); huhao07@nudt.edu.cn (H.H.); chlguan@nudt.edu.cn (C.G.); d20153105@163.com (Z.L.); 2 Hunan Key Laboratory of Ultra-Precision Machining Technology, Changsha 410073, China

**Keywords:** edge effect, combined polishing method, CCOS

## Abstract

The computer-controlled optical surface (CCOS) can process good optical surfaces, but its edge effect greatly affects its development and application range. In this paper, based on the two fundamental causes of the CCOS’s edge effect—namely the nonlinear variation of edge pressure and the unreachable edge removal—a combined polishing method of double-rotor polishing and spin-polishing is proposed. The model of the combined polishing method is established and theoretically analyzed. Combined with the advantages of double-rotor polishing and spin-polishing, the combined polishing process can achieve full-aperture machining without pressure change. Finally, the single-crystal silicon sample with a diameter of 100 mm is polished by the combined polishing process. The results show that, compared with the traditional CCOS polishing, the residual error of the sample after the combined polishing process is more convergent, and the edge effect is effectively controlled.

## 1. Introduction

Computer-controlled optical surfacing technology has been widely used for the ultra-precision machining of various optical materials and plays a pivotal role in this process. However, as with micro-milling, the further development of CCOS is severely limited by the edge effect in the processing [1,2,3]. The edge effect is mainly caused by two reasons: first, the edge area of the workpiece cannot be reached by the orbital motion of the polishing disc; and second, the non-linear variation of the pressure at the edge of the workpiece leads to the inaccuracy of the tool influence function (TIF) [4,5,6,7].

Many scholars have conducted in-depth studies to address these problems. Various TIF algorithm models have been proposed to simulate and calibrate the variations of actual TIF at the edges. Among the early representative theories are the linear pressure distribution model by Wagner [8] and the skin model by Luna-Aguilar [9]. A new edge pressure model is developed based on the results of finite element analysis. The basic pressure distribution can be calculated based on the surface shape of the polishing pad, a correction function is used to compensate for the errors caused by edge effects, and the edge TIF with different overhang rates can be accurately predicted [5,10]. Surveys such as that conducted by W. Song [11] have shown that the generalized spatial variable deconvolution algorithm can accurately calculate the dwell time, which can better control the actual removal amount so as to effectively suppress the edge error and improve the convergence rate. Based on the errors distribution on the workpiece, Yu et al. [12] developed a new tool running path, which not only reduces residual errors on the edges but also the total polishing time.

On the other hand, a considerable amount of literature has been published on how to obtain an eccentric TIF. The purpose of these studies is to expand the scope of the actual processing as much as possible, even full-aperture processing. A polishing method based on surface extension is proposed. By simulating the pressure distribution of the workpiece under different overhangs, the exact removal function under different overhangs is obtained, and the optimal parameters that can effectively suppress the edge effect are obtained [13]. Hongyu-Li established a novel edge-control technique based on “progressive” polishing technology, which obtained an accurate and stable edge tool influence function (TIF) and low residual surface errors [14,15]. In 2016, a new concept of the ‘heterocercal’ tool influence function (TIF) was developed by Haixing-Hu [16], which was generated from compound motion equipment. This type of TIF can better remove the edge area of the sample. In addition, it also has high removal efficiency and surface quality. In 2017, Hang-Du [17] reported an acentric tool influence function (A-TIF) was designed to suppress the rolled edge after CCOS polishing. It has been proven to be effective through experiments.

The above-mentioned work largely suppressed the edge effect of CCOS polishing, but there are certain limitations to solving the two fundamental causes of the edge effect. In this paper, by combining the advantages of double-rotor polishing and spin-polishing, a combined polishing process is proposed. It aims to solve the two fundamental problems mentioned above simultaneously and provides a new way to control the edge effect of CCOS.

## 2. Theory of Combined Polishing Process

### 2.1. Basic Polishing Theory

Define the removal function, *R* (*x*, *y*), as the average amount of material removed per unit time by a tool that does not move. The basic principle of CCOS polishing is Preston’s equation [18], the TIF of which can be calculated based on the equation of material removal, as shown in Equation (1).

(1) $$R\left(x,y\right)=\frac{\Delta Z\left(x,y\right)}{T}=\frac{1}{T}\int_{0}^{T}k_{0}P\left(x,y\right)V\left(x,y\right)dt.$$

Here, ∆Z (*x*, *y*) is the total amount of material removed from the workpiece, *P* (*x*, *y*) is the pressure of the tool on the workpiece, *V* (*x*, *y*) is the relative velocity between the tool and the workpiece, and *T* is the dwell time. *k*_0_ is the Preston coefficient, which is related to the processing temperature, polishing fluid, and other processing conditions.

**Figure 1:**
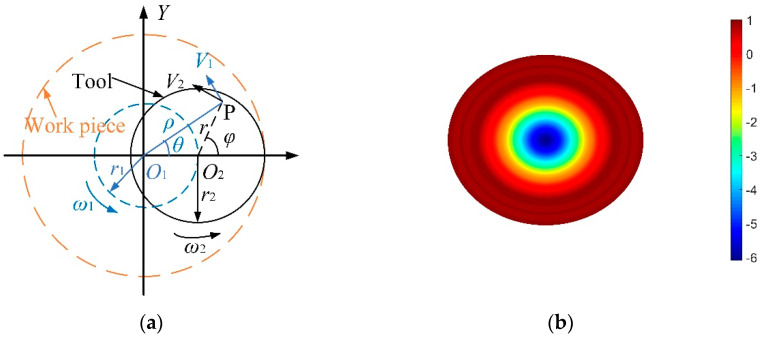
Theory of double-rotor polishing, (**a**) motion analysis of double-rotor polishing, (**b**) simulation of TIF.

Figure 1a shows a schematic diagram of two velocity fields generated from orbital motion *V*_1_ and spin motion *V*_2_. P is any point in the overlap area of the sample and tool. *r*_1_ is the offset of tool center *O*_2_ relative to rotation center *O*_1_ and *r*_2_ is the radius of the tool. ω_1_ and ω_2_ are the orbital angular velocity and spin angular velocity of the tool, respectively. The total velocity, *V*, can be expressed as Equation (2).

(2) $$\left\{ \begin{array}{l} V^{2}=V_{x}{}^{2}+V_{y}{}^{2}\\ V_{x}=-V_{1}\sin\theta -V_{2}\sin\varphi \\ V_{y}=V_{1}\cos\theta +V_{2}\cos\varphi \end{array}\right..$$

Assuming $f=\omega_{2}/\omega_{1}$, $e=r_{2}/r_{1}$, combining Equations (1) and (2), the TIF of double rotor polishing, *R*_2_ (*x*, *y*), can be expressed as [19]:

(3) $$\left\{ \begin{array}{l} R_{2}\left(x,y\right)=\frac{kP\left(x,y\right)\omega_{1}}{2\pi }{\int_{-\theta_{0}}^{\theta_{0}}\left[\rho^{2}\left(1+f^{2}\right)+r_{2}{}^{2}f^{2}e^{2}-2\rho r_{2}fe\left(1+f\right)\cos\theta \right]}^{\frac{1}{2}}d\theta \\ \rho \in \left[0,\left(1+e\right)r_{2}\right],\theta_{0}=\arccos\left(\frac{\rho^{2}+\left(e^{2}-1\right)r_{2}{}^{2}}{2\rho er_{2}}\right) \end{array}\right..$$

According to Equation (3), a TIF simulation of the double-rotor polishing with a Gaussian-like shape is shown in Figure 1b. This type of TIF has high removal efficiency for the intermediate area and low removal efficiency for the edge area, which can provide good processing capability for polishable areas. In addition, different height values are indicated by different colors, while the numbers next to them indicate relative heights.

**Figure 2:**
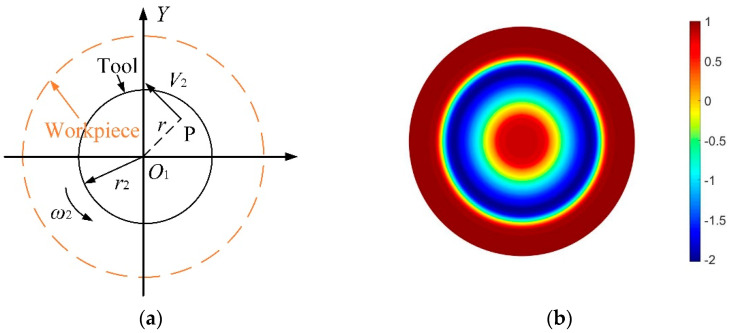
Theory of spin-polishing, (**a**) motion analysis of spin-polishing, (**b**) simulation of TIF.

Figure 2a shows the motion analysis of the spin-polishing, whose total speed *V* is equal to the tool’s spin speed *V*_2_, as shown in Equation (4). Combining Equations (1) and (4), the TIF of spin-polishing can be obtained, as shown in Equation (5). Figure 2b shows the TIF simulation of the spin-polishing. This TIF is W-shaped, and it has high removal efficiency for the edge area and low removal efficiency for the intermediate area, which is contrary to the characteristics of double-rotor polishing.

(4) $$V=V_{2}=r\omega_{2}.$$

(5) $$\left\{ \begin{array}{l} R_{1}\left(x,y\right)=\int_{0}^{T}kP\left(x,y\right)\cdot r\omega_{2}dt\\ r\in \left(0,r_{2}\right) \end{array}\right..$$

**Figure 3:**
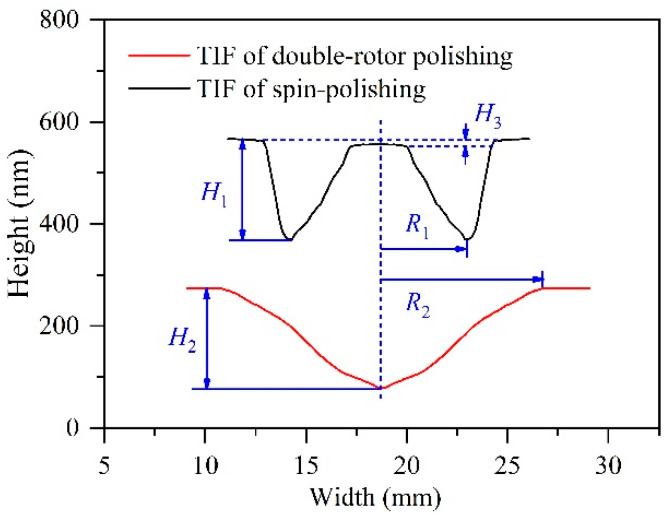
Cross-sectional view of TIF.

The TIF’s cross-sectional profiles of the double-rotor polishing and spin-polishing are shown in Figure 3. As seen in the graph, it is clear that the distribution of the two TIFs’ removal peaks is highly complementary. That is, where the double-rotor polishing removal is high the spin-polishing removal is low, and vice versa. *H*_1_ and *H*_2_ are the peak height removed by spin-polishing and double-rotor polishing, respectively. Theoretically, the removal rate at the center of the spin-polishing’ TIF is 0. However, due to the effect of long-time pressure and the accuracy of machine movement, a very small removal amount, *H*_3_, occurs here. The effective radius *R*_1_ of the spin-polishing TIF is equal to the tool’s radius *r*_2_, whereas the double-rotor polishing’s effective radius *R*_2_ is the sum of the tool’s radius *r*_2_ and offset *r*_1_, as shown in Equation (6).

(6) $$\left\{ \begin{array}{l} R_{1}=r_{2}\\ R_{2}=r_{1}+r_{2} \end{array}\right..$$

### 2.2. Combined Polishing Method

In the CCOS polishing process, the tool moves over the surface of the workpiece following a predetermined trajectory and stays at each arbitrary point for a certain time. The material removed by the tool in each area of the workpiece surface can be superimposed together to obtain the distribution function of the surface errors. In other words, the distribution function of the surface errors is equal to the convolution of the removal function and the dwell time, *D* (*x*, *y*), as shown in Equation (7).

(7) $$H\left(x,y\right)=\iint_{A}R\left(x-\alpha ,y-\beta \right)D\left(\alpha ,\beta \right)d\alpha d\beta =R\left(x,y\right)\times D\left(x,y\right).$$

It is well known that the overhang of the tool will lead to a non-linear variation in pressure, which is an overwhelming factor causing edge effects. So, what would happen if there were no overhang of the tools? Although the width of the rolled edge is increased, the accuracy of CCOS polishing is also improved. Therefore, in order to eliminate the influence of nonlinear pressure and a collapsed edge, the combined polishing process is all based on no overhang of tools. The basic ideas of the combined polishing process are: (1) Minimizing the height of the rolled edge as much as possible when using non-overhanging double-rotor polishing; (2) Minimizing the width of the rolled edge using the minimal tool; (3) Reducing the height of the rolled edge with the commutative method of the spin-polishing; (4) Repairing of annular residual errors caused by spin-polishing using the double-rotor polishing method and finally obtaining a flawless surface.

**Figure 4:**
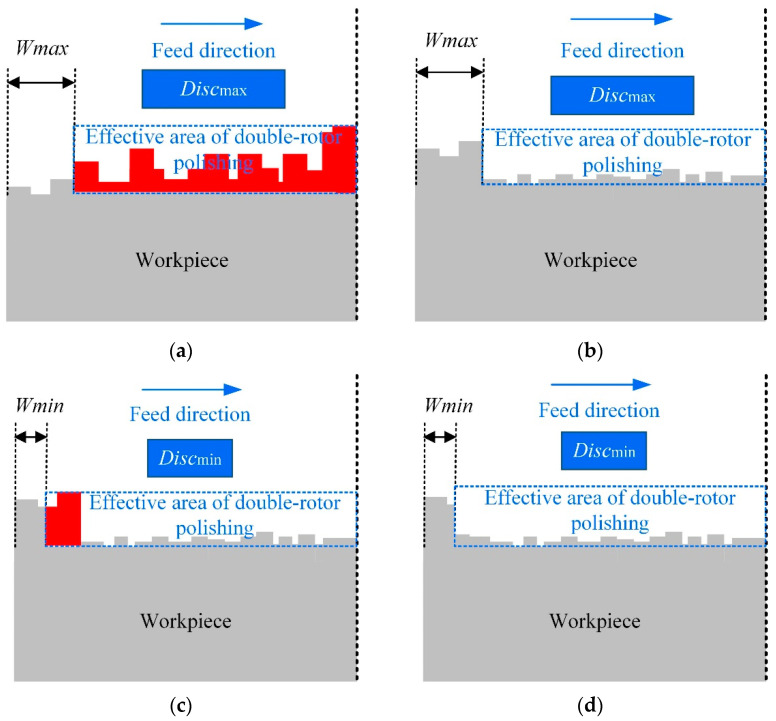
Edge removal theory of double-rotor polishing. (**a**) initial surface profile and area to be removed for the first time, (**b**) surface profile after polishing with a large tool, (**c**) area to be removed for the second time, (**d**) surface profile after polishing with a small tool.

When CCOS is used to polish the workpiece, to a certain extent the rolled edge will inevitably occur, as shown in Figure 4a,b. Therefore, the combined polishing process proposed in this paper was used to solve this problem. Firstly, a large tool is used to polish the workpiece quickly and efficiently. At the same time, a safety factor *K* ∈ (0, 1) is introduced to control the height of the rolled edge, which can prevent over-processing. However, since there is no overhang of the tool during the polishing process, a large unmachined area *W*_max_ emerged at the edge of the workpiece. Second, since the small tool has a small TIF, the width of the unmachined area at the edge of the workpiece can be reduced, as shown in Figure 4c,d. When the red area in the figure is removed by the small tool, the width of the edge unmachined area is reduced from *W*_max_ to *W*_min_, which can eventually be reduced to less than 10 mm. It is worth noting that the effective area of the double-rotor polishing does not need to be particularly flat at this point. This can provide a processing allowance for subsequent spin-polishing to remove the height of the rolled edge.

**Figure 5:**
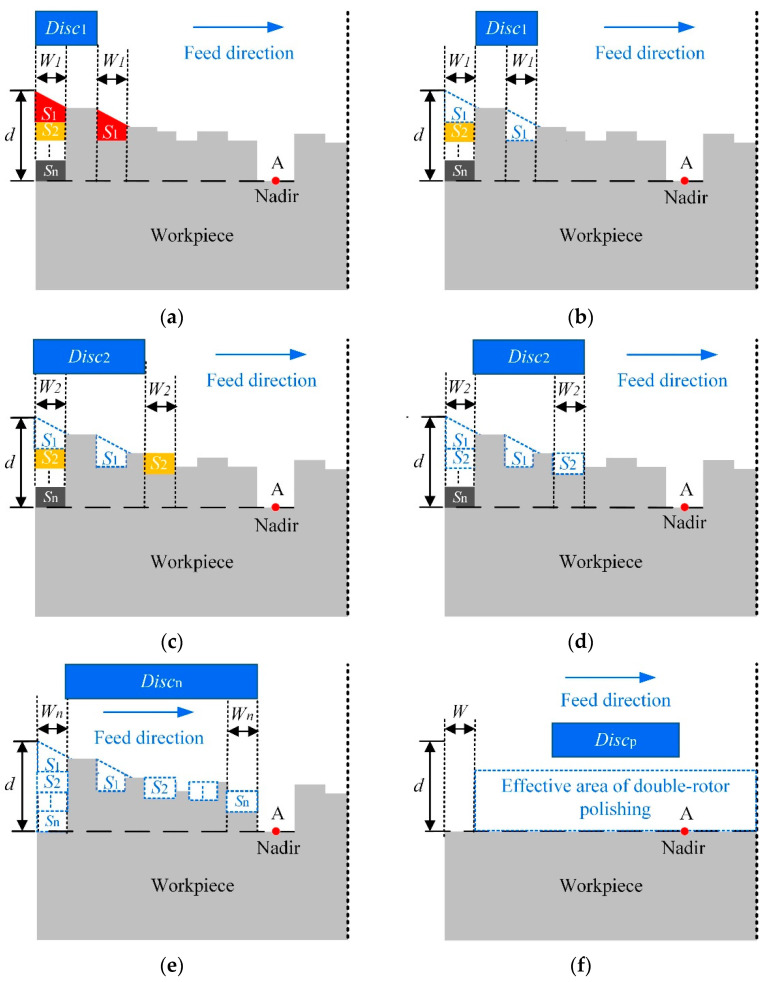
Edge removal theory of the combined polishing. (**a**) initial surface profile, (**b**) surface profile after the first spin-polishing, (**c**) surface contours before the second spin-polishing, (**d**) surface profile after the second spin-polishing, (**e**) surface profile after the n-th spin-polishing, (**f**) surface profile after the double-rotor polishing.

After minimizing the width of the rolled edge, the most important thing is how to decrease the height of the rolled edge. The detailed method of using spin-polishing to remove the height of the rolled edges is shown in Figure 5. The core idea of this method is the equivalent replacement. Firstly, the number n of polishing discs of different sizes is determined based on the measured surface error distribution of the workpiece. Based on the basic principle that the height of the middle area after being removed is not lower than the lowest point A of the full aperture, the height of the rolled edge is divided into n segments that match the surface error of the workpiece, as shown in Figure 5a. *d* is the height from the highest point of rolled edge to the lowest point of full aperture, which is divided into n regions, such as *S*_1_, *S*_2_, …, *S*_n_. *W_i_* and *Disc_i_* are the width and the polishing tool used of the corresponding area, respectively. Second, the distribution function of the surface error *H_i_* (*x*, *y*) needs to be calculated exactly before each polishing. *H_i_* (*x*, *y*) is the sum of the distribution function of *S*_i_ and the distribution function of the other areas of the full aperture excluding *S_i_*. The distribution function of the total removal *H* (*x*, *y*) is then equal to the sum of the distribution functions for each polishing. Their relationships are shown in Equations (8) and (9). Combined with the removal efficiency of the removal function, the distribution of the residence time of each polish can be obtained.

(8) $$\left\{ \begin{array}{l} H\left(x,y\right)=\sum_{1}^{n}H_{i}\left(x,y\right)\\ H_{i}\left(x,y\right)=H_{S_{i}}\left(x,y\right)+H_{\left(A_{i}-S_{i}\right)}\left(x,y\right) \end{array}\right.\left(i=1,2\cdots n\right).$$

(9) $$\left\{ \begin{array}{l} H_{S_{i}}\left(x,y\right)=\iint_{S_{i}}R\left(x-\alpha ,y-\beta \right)D\left(\alpha ,\beta \right)d\alpha d\beta \\ H_{\left(A_{i}-S_{i}\right)}\left(x,y\right)=\iint_{\left(A_{i}-S_{i}\right)}R\left(x-\alpha ,y-\beta \right)D\left(\alpha ,\beta \right)d\alpha d\beta \end{array}\right.\left(i=1,2\cdots n\right).$$

Here *S_i_* is the area of rolled edge to be removed in the *i*-th polishing, *A_i_* is the full-aperture zone before the *i*-th polishing. (*A_i_*-*S_i_*) is the other regions of the full aperture excluding *S_i_*. *H_Si_* (*x*, *y*) is the distribution function of the surface material to be removed in *S_i_* for the *i*-th polishing, and *H*_(*Ai*-*Si*)_ (*x*, *y*) is the distribution function of the surface material to be removed in (*A_i_*-*S_i_*) for the *i*-th polishing.

In addition, after the distribution function of the removal amount of the spin-polishing is determined, the rolled edges can be removed iteratively using the combined polishing method and finally eliminated, shown in Figure 5a–e. Assume that the initial surface profile is shown in Figure 5a. The area *S*_1_ is removed in the first spin-polishing and the machined surface profile is obtained, as shown in Figure 5b. Similarly, the surface contours before and after the second polishing are shown in Figure 5c,d, respectively. Then, the rolled edge can be completely eliminated theoretically after n iterations, as shown in Figure 5e. After the elimination of the rolled edge, the intermediate area can be polished with precision by selecting a suitable size tool, and finally, a high-quality surface is obtained, as shown in Figure 5f.

Moreover, in order to realize the assumptions of the combined polishing process and to improve the convergence rate of the surface errors, some parameters also need to be constrained, as shown in Equation (10). This ensures that the edge removal width is greater than the width of the rolled edge and that the middle equivalent removal zones do not overlap as much as possible. Here, *W*_min_ is the width of the rolled edge after polishing with the smallest tool, *W_i_* is the width of the rolled edge to be removed by the *i*-th polishing. *D_i_* is the diameter of the tool used for the *i*-th polishing, *D_i_*_+ 1_ is the diameter of the tool used for the (*i* + 1)-th polishing.

(10) $$\left\{ \begin{array}{l} W_{i}\geq W_{\min}\\ D_{i+1}\geq D_{i}+W_{i} \end{array}\right.\left(i=1,2\cdots n\right).$$

**Figure 6:**
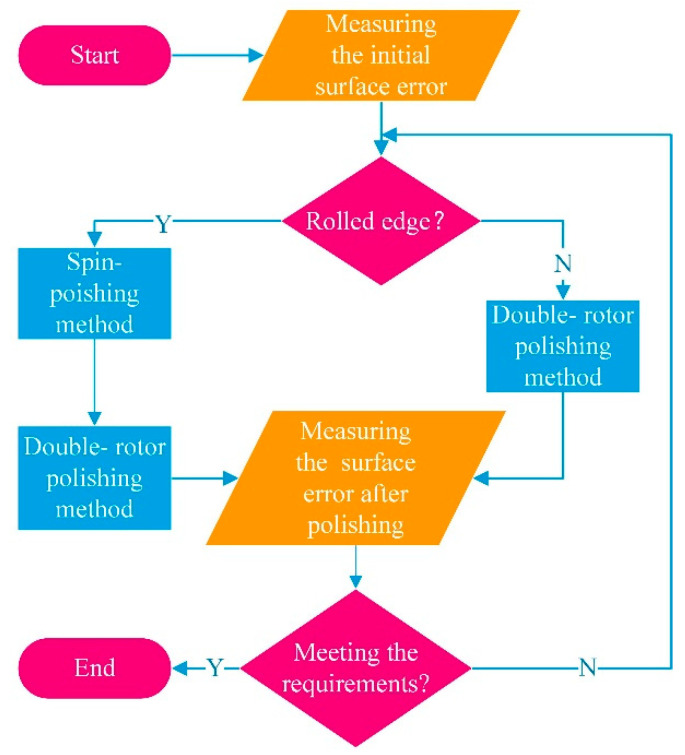
Combined polishing method.

Figure 6 shows the whole flow chart of the combined polishing process. The first thing is to measure the initial surface error of the workpiece. Then, the choice of polishing process is determined by whether the surface error has a rolled edge or not. If it does, the spin-polishing is preferred to reduce the height of the rolled edge. Then the double-rotor polishing method without overhang is used to polish the middle area of the workpiece. Lastly, the polished surface is inspected and judged on whether it meets the requirements. On the other hand, if there is a collapsed edge, it is processed directly by double-rotor polishing. In conclusion, the combined polishing process not only avoids the nonlinear variation of edge pressure but also solves the problem of unreachable trajectory, which has an excellent effect on the control of edge effect.

## 3. Edge Processing Experiment

In order to verify the credibility and feasibility of the aforementioned combined polishing process, a single crystal silicon sample was selected for the polishing experiments. The specific experimental parameters are shown in Table 1. The detailed experiment is as follows: First, a polishing tool with a diameter of 30 mm was used for polishing, which could quickly remove material. Then, a polishing tool with a diameter of 10 mm was used to reduce the width of the rolled edge. After that, three sizes of polishing tools were used to reduce the height of the rolled edge. Finally, a 20 mm polishing disc was used for reshaping.

## 4. Results and Discussion

**Figure 7:**
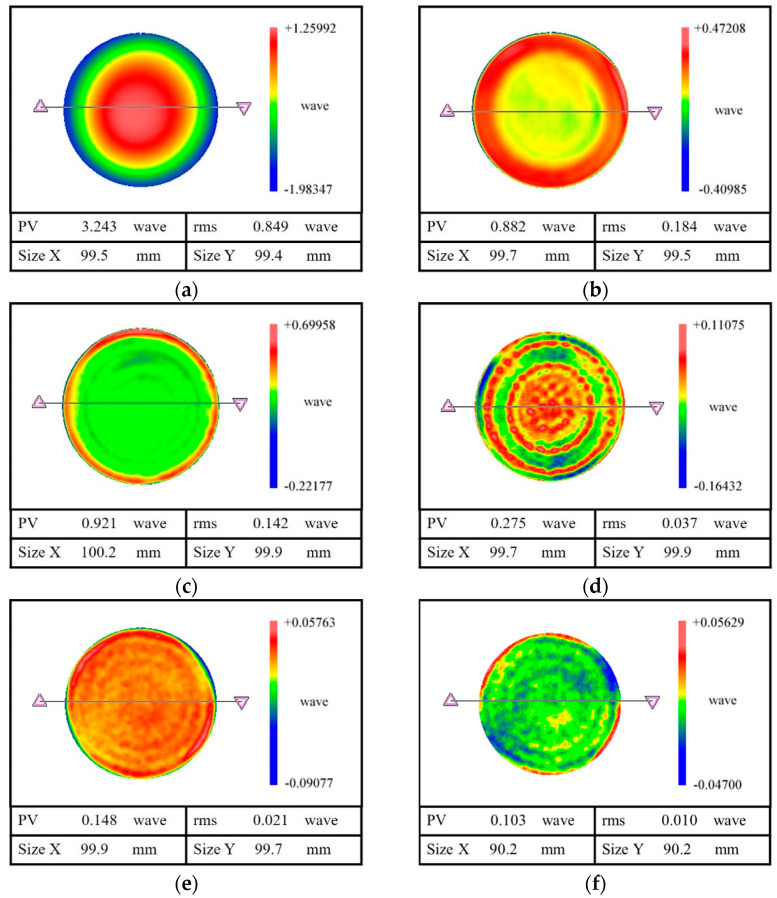
Surface error after combined polishing process. (**a**) initial surface errors, (**b**) surface errors after the double–rotor polishing with large tool, (**c**) surface errors after the double–rotor polishing with a small tool, (**d**) surface errors after the spin–polishing, (**e**) surface errors after the combined polishing method, (**f**) surface errors of 90% area after the combined polishing method.

The initial surface error of the sample is 3.243λ PV, 0.849λ RMS and is shown in Figure 7a. Since the initial surface error is the collapsed edge, a double-rotor polishing method with a large tool was first chosen for polishing. Then, a figure accuracy of 0.882λ PV, 0.184λ RMS was obtained and is shown in Figure 7b. Compared to the initial surface error, the residual surface error after polishing is greatly converged. Moreover, it can be found that although the surface error in the middle region is converged, there are clear rolled edges appearing at the edges of the specimen. Then the double-rotor polishing method with the smallest tool was employed to reduce the width of the rolled edges. The surface error with 0.921λ PV, 0.142λ RMS was obtained, as shown in Figure 7c. Compared with the surface error in Figure 7b, the width of the rolled edges is significantly reduced. With this, the suppression of rolled edges’ width in the combined polishing process is realized.

According to the aforementioned combined polishing method, the spin-polishing method was selected to reduce the height of the rolled edge. The surface error after spin-polishing is a ring band of varying heights, as shown in Figure 7d. Its PV decreases from 0.921λ to 0.275λ, and RMS decreases from 0.142λ to 0.037λ, which obviously improves the surface quality of the sample. What is more noteworthy is that the height of the rolled edge is significantly reduced. After the combined polishing process, the sample with a surface accuracy of 0.148λ PV and 0.021λ RMS is finally obtained, as shown in Figure 7e. In addition, it is worth noting that a figure accuracy of 0.103λ PV, 0.010λ RMS can be obtained in 90% of the area after the combined polishing. Compared with the conventional CCOS polishing, such as Figure 7b,c, the edge effect of the sample is greatly weakened after the combined polishing process, and the surface quality is greatly improved. The results show that the aforementioned combined polishing process is of great significance for controlling the edge effect of CCOS polishing, which also verifies the effectiveness and practicality of the combined polishing process.

**Figure 8:**
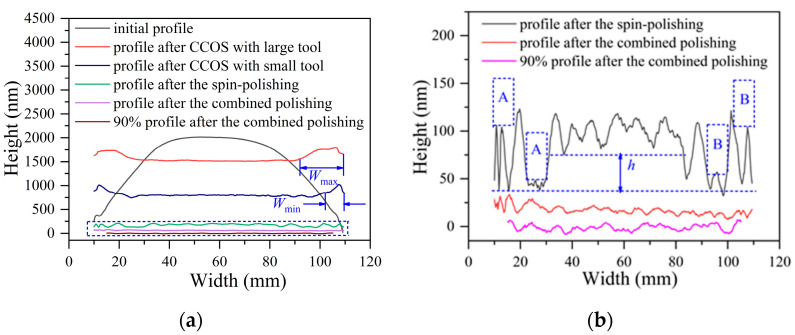
Surface profile after different machining processes. (**a**) profile comparison at different processing stages, (**b**) amplified view of the dashed part in (**a**).

The profiles at different stages of the combined polishing process are shown in Figure 8a. What is clear is that the profile after double-rotor polishing with a large tool has a larger width of the rolled edge *W*_max_, as the effective radius of the double-rotor polishing. In other words, when machining with a small tool, the width *W*_max_ of the rolled edge will be gradually reduced until the minimum value *W*_min_. At this point, the first step of the combination polishing is completed, namely, reducing the width of the rolled edge.

An amplified view of the dashed area in Figure 8a is shown in Figure 8b. From the profile after spin-polishing, the surface residual error is in line with the expectation of the combined polishing process. In this case, the height of the rolled edge is reduced by removing the height of the middle area simultaneously, as in regions A and B in Figure 8b. This shows that the method of reducing the height of the rolled edge by spin-polishing is feasible. However, special attention should be paid to the accurate calculation of the height to be removed for each polishing before processing. The best result is that the height of the intermediate region after polishing is equal to the minimum height of the initial surface, that is, *h* is 0. This helps to reduce the amount of subsequent processing and the convergence rate of surface errors. Comparing the surface profile before and after the combined polishing process, the results show that the combined polishing process is very effective in suppressing the edge effect of CCOS.

## 5. Conclusions

In summary, a combined polishing process was proposed for the edge effects that occur in CCOS. In addition, a theoretical analysis and experimental validation of the combined polishing process were carried out. During the combined polishing process, the pressure variation was limited and the edge area was effectively removed. Finally, the PV value of the surface error was reduced from 3.243λ to 0.148λ, which indicates that the combined polishing process has a good suppression effect on the edge effect.

## Figures and Tables

**Table 1:** Experimental parameters.

Category	Parameter
The material of the polishing pad	Polyurethane
The size of tools	ϕ10, 20 and 30 mm
Polishing slurry	Silica(Φ50 nm)
Pressure	0.06~0.1 MPa
Rotating speed	80~120 r/min

## Data Availability

The data presented in this study are available on request from the corresponding author. The data are not publicly available due to the data also forms part of an ongoing study.

## References

[1] Chen Y., Wang T., Zhang G. (2020). Research on Parameter Optimization of Micro-Milling Al7075 Based on Edge-Size-Effect. Micromachines.

[2] Deja M. (2010). Simulation Model for the Shape Error Estimation During Machining With Flat Lapping Kinematics, ASME 2010. Int. Manuf. Sci. Eng. Conf..

[3] Zhang X.H., Pei Z.J., Fisher G.R. (2006). A grinding-based manufacturing method for silicon wafers: Generation mechanisms of central dimples on ground wafers. Int. J. Mach. Tools Manuf..

[4] Huang J., Hu Q., Xie L., Ma P. Research on surface extension process technology for restraining edge effect in CNC polishing. Proceedings of the Second Target Recognition and Artificial Intelligence Summit Forum.

[5] Liu H., Wu F., Zeng Z., Fan B., Wan Y. (2014). Edge effect modeling and experiments on active lap processing. Opt. Express.

[6] Lu A., Jin T., Guo Z., Qu M., Chang Y., Liu Q., Zhang C. (2018). Characterization of the tool influence function in a dual-axis wheel polishing process to achieve high material removal rates. Precis. Eng..

[7] Walker D., Beaucamp A., Dunn C., Evans R., Freeman R., Morton R., Wei S., Yu G. (2008). Active control of edges and global microstructure on segmented mirrors. Advanced Optical and Mechanical Technologies in Telescopes and Instrumentation.

[8] Wagner R., Shannon R. (1974). Fabrication of aspherics using a mathematical model for material removal. Appl. Opt..

[9] Luna-Aguilar E., Cordero-Davila A., Gonzalez J., Nunez-Alfonso M., Cabrera V., Robledo-Sanchez C.I., Cuautle-Cortez J., Pedrayes M.H. (2003). Edge effects with the Preston equation. Future Giant Telescopes.

[10] Nam H.-S., Kim G.-C., Kim H.-S., Rhee H.-G., Ghim Y.-S. (2016). Modeling of edge tool influence functions for computer controlled optical surfacing process. Int. J. Adv. Manuf. Technol..

[11] Wan S., Zhang X., Wang W., Xu M., Jiang X. (2019). Edge control in precision robotic polishing based on space-variant deconvolution. Precis. Eng..

[12] Yu G., Walker D., Li H., Zheng X., Beaucamp A. (2017). Research on edge-control methods in CNC polishing. J. Eur. Opt. Soc. Rapid Publ..

[13] Ke X., Qiu L., Wang C., Wang Z. (2020). Tentative Investigations on Reducing the Edge Effects in Pre-Polishing the Optics. Appl. Sci..

[14] Li H., Walker D., Yu G., Sayle A., Messelink W., Evans R., Beaucamp A. (2013). Edge control in CNC polishing, paper 2: Simulation and validation of tool influence functions on edges. Opt. Express.

[15] Walker D., Yu G., Li H., Messelink W., Evans R., Beaucamp A. (2012). Edges in CNC polishing: From mirror-segments towards semiconductors, paper 1: Edges on processing the global surface. Opt. Express.

[16] Hu H., Zhang X., Ford V., Luo X., Qi E., Zeng X., Zhang X. (2016). Edge control in a computer controlled optical surfacing process using a heterocercal tool influence function. Opt. Express.

[17] Du H., Song C., Li S., Xu M., Peng X. (2017). Optimization technique for rolled edge control process based on the acentric tool influence functions. Appl. Opt..

[18] Preston F. (1927). The theory and design of plate glass polishing machines. J. Glass Technol..

[19] Xusheng Z., Shengyi L., Ziwen Z. (2005). Optimization of Plane Polishing Parameters Based on Maximum Entropy Principle. Chin. Mech. Eng..

